# Supplementary material for: Coherent electrical readout of defect spins in silicon carbide by photo-ionization at ambient conditions
Source: Nat Commun. 2019 Dec 5;10:5569. doi: 10.1038/s41467-019-13545-z (PMC6895084; doi:10.1038/s41467-019-13545-z)
Supplement: Supplementary file 1 — Supplementary Information [file 41467_2019_13545_MOESM1_ESM.pdf]

Supplementary Information: Coherent electrical readout of defect spins in silicon carbide by photo-ionization at ambient conditions

Matthias Niethammer,[1,*] Matthias Widmann,[1] Torsten Rendler,[1] Naoya Morioka,[1] Yu-Chen Chen,[1] Rainer Stöhr,[1] Jawad Ul Hassan,[2] Shinobu Onoda,[3] Takeshi Ohshima,[3] Sang-Yun Lee,[4] Amlan Mukherjee,[1] Junichi Isoya,[5] Nguyen Tien Son,[2] and Jörg Wrachtrup[1,6]

[1]*3rd Institute of Physics and Center for Applied Quantum Technologies, University of Stuttgart, 70569 Stuttgart, Germany*

[2]*Department of Physic, Chemistry and Biology, Linköping University, SE-581 83 Linköping, Sweden*

[3]*National Institutes for Quantum and Radiological Science and Technology, Takasaki 370-1292, Japan*

[4]*Center for Quantum Information, Korea Institute of Science and Technology, Seoul 02792, Republic of Korea*

[5]*Faculty of Pure and Applied Sciences, University of Tsukuba, Tsukuba 305-8573, Japan*

[6]*Max Planck Institute for Solid State Research, 70569 Stuttgart, Germany*

(Dated: November 11, 2019)

Supplementary Method I: Experimental setup and measurement schemes

The experimental setup consists of a home-built scanning optical microscope capable of detecting both optical and electrical signals to perform spin resonance measurements as shown in Supp. Fig. 1.

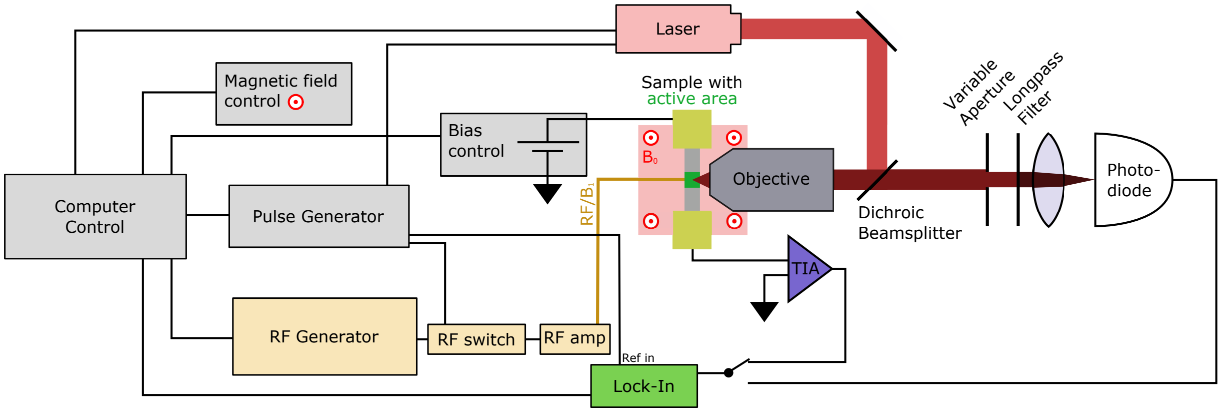

Supplementary Figure 1. Sketch of the experimental setup.

For excitation, a 785 nm laser is coupled into a NA 0.65 microscope objective using a 810 nm dichroic beamsplitter. For fluorescence detection, the emission is collected by the same objective, transmitted through the dichroic beamsplitter and finally filtered by a 850 nm cut-off long-pass filter before it is focused onto a Si photodetector. Additionally, a variable aperture is placed before the detector in order to reduce the fluorescence light on the photodiode and provide larger dynamic range. This is needed to ensure that the same conditions in terms of laser power and duty cycle can be used in the optical as well as electrical detected measurements without overloading the detector.

The spin-driving radio-frequency field is created by a signal generator, modulated by a fast microwave switch and amplified by a radio frequency (RF) amplifier. It is then fed to the device structure *via* a coplanar wave-guide below the sample. Additionally, an offset magnetic field B_0 can be applied by a home-built 3D Helmholtz coil assembly, in which the sample is centered. The coil arrangement allows for up to $\approx 70\,\mathrm{G}$ magnetic field along the crystal c-axis.

The photocurrent extraction is performed by applying a bias voltage to one of the contacts

while the second device contact is connected to a transimpedance amplifier, which converts the photocurrent to voltage with a gain of 10^8 (PDMR) or 10^9 (Rabi, Hahn echo) before feeding the signal into a lock-in amplifier.

Both fluorescence and photocurrent are detected using a lock-in based measurement. The spin control pulse sequence, the laser control pulses and the lock-in reference are all synchronized and created by a home-built FPGA-based pulse generator.

The pulse sequences used are shown in Supp. Fig. 2 and are shortly explained in the following.

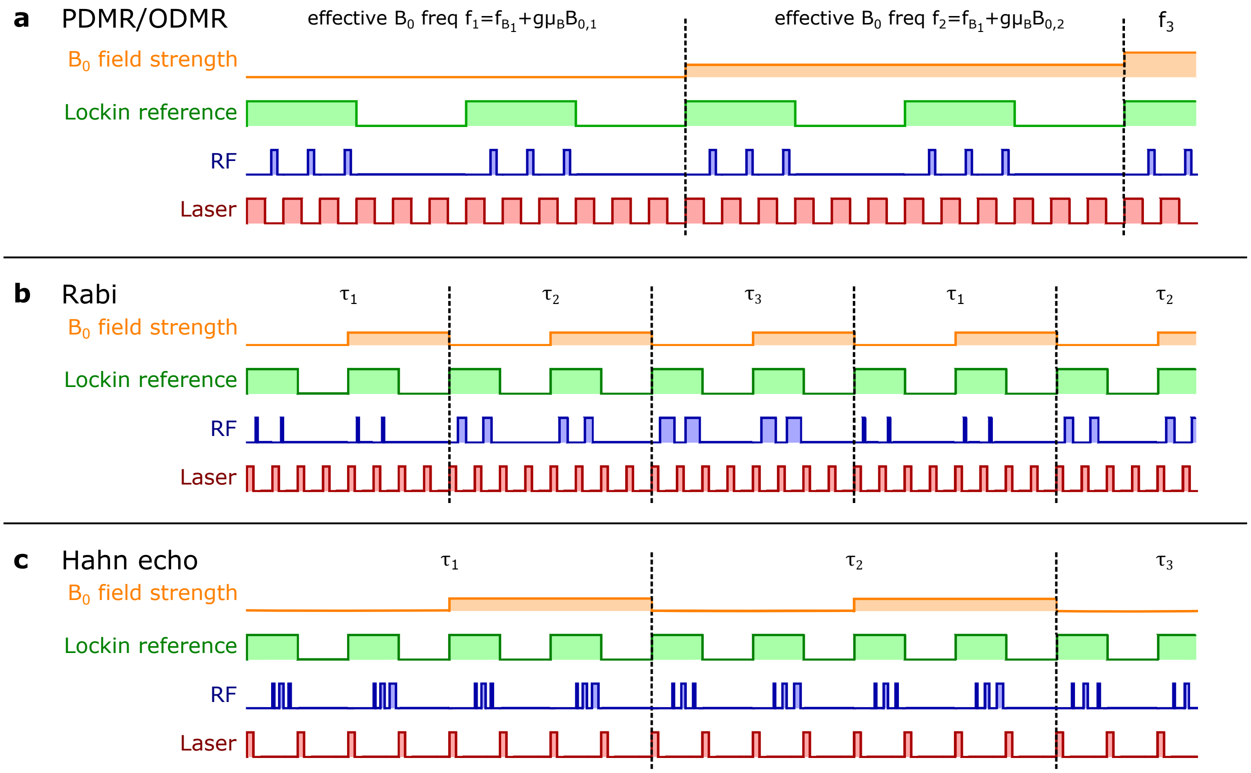

Supplementary Figure 2. Pulse sequences and measurement scheme used. **a** For PDMR B_0 field strength is altered for an effective frequency sweep, while the lockin sequence performes a pulsed ESR measurement. **b** Rabi measurement scheme performs a Rabi sequence at on- and off-resonance and subtracted for canceling out effects of the varying RF pulse length **c** Hahn echo is performed at on-and off-resonance condition as well as with $\pi/2$-π-$\pi/2$ and $\pi/2$-π-$3\pi/2$ sequences in order to obtain the spin signal. In all cases, lock-in is achieved by modulating the RF sequence on and off at the lock-in frequency.

The pulse sequence for the ODMR and PDMR measurements (Supp. Fig. 2**a**) uses a first

laser pulse to initialize the system followed by an RF π-pulse to flip the spins. The next laser pulse reads out the spin state. This sequence is repeated multiple times, followed by a run without the RF pulse. The RF-on and RF-off sequence are switched synchronous with the lock-in reference pulses at a frequency of ≈449 Hz in order to lock the lock-in amplifier and detect the change in signal created due to spin driving. By altering only the B_0 field, and averaging several acquisitions at each step we effectively perform an RF frequency sweep for the spin system. This removes background contribution from the RF-frequency dependent coupling to the device and thus improves SNR.

The Rabi signal is obtained by the sequence shown in Supp. Fig. 2**b**. After the first initializing laser pulse, an on-resonance RF pulse of variable length drives the spin. This sequence is repeated multiple times within one lock-in half-period. Afterwards, the sequence is repeated without applied RF drive to produce the second half-period of the lock-in. This full sequence is then acquired at a different off-resonant frequency-magnetic field combination and subtracted from the on-resonance signal, in order to remove influence of the varying RF pulse width from the signal.

Finally, Supp. Fig. 2**c** sketches the pulse sequence used for the Hahn echo measurements. The sequence consists of the initializing laser pulse followed by a $(\pi/2)$-$(\tau/2)$-(π)-$(\tau/2)$-$(\pi/2)$ sequence at an on-resonant frequency-field condition. The signal is obtained by repeating the laser sequence without microwave in order to lock to the spin signal. After a few accumulations, the sequence is then repeated with a $(\pi/2)$-$(\tau/2)$-(π)-$(\tau/2)$-$(3\pi/2)$ RF pulse. Here, the duty cycle is kept constant by prolonging the off-time between the last $\pi/2$ in part one and the following laser pulse. Both sequences are repeated at an off-resonant condition. The signal for $(\pi/2)$ and $(3\pi/2)$ is then obtained by subtracting the off-resonant accumulations from the on-resonant ones. Finally, to obtain the spin signal, the $(\pi/2)$-signal is subtracted from the $(3\pi/2)$-signal.

The lock-in frequency of 429 Hz is chosen such that the limited bandwidth of the photodiode (750 Hz) and transimpedance amplifier (1 kHz) do not damp the signal and are not a multiple of the power line frequency 50 Hz.

Supplementary Method II: Sample growth and fabrication details

Different layers were grown by chemical vapor deposition (CVD) on 4° off-axis n-type 4H-SiC substrate. The first layer grown is a 10 µm thick semi-insulating V-doped layer followed by a n^- layer (10 µm) with a free carrier concentration of $1 \times 10^{14}\,\mathrm{cm}^{-3}$ at room temperature. The top n^{++}-type N-doped contact layer is 400 nm thick with a doping concentration of $8 \times 10^{17}\,\mathrm{cm}^{-3}$.

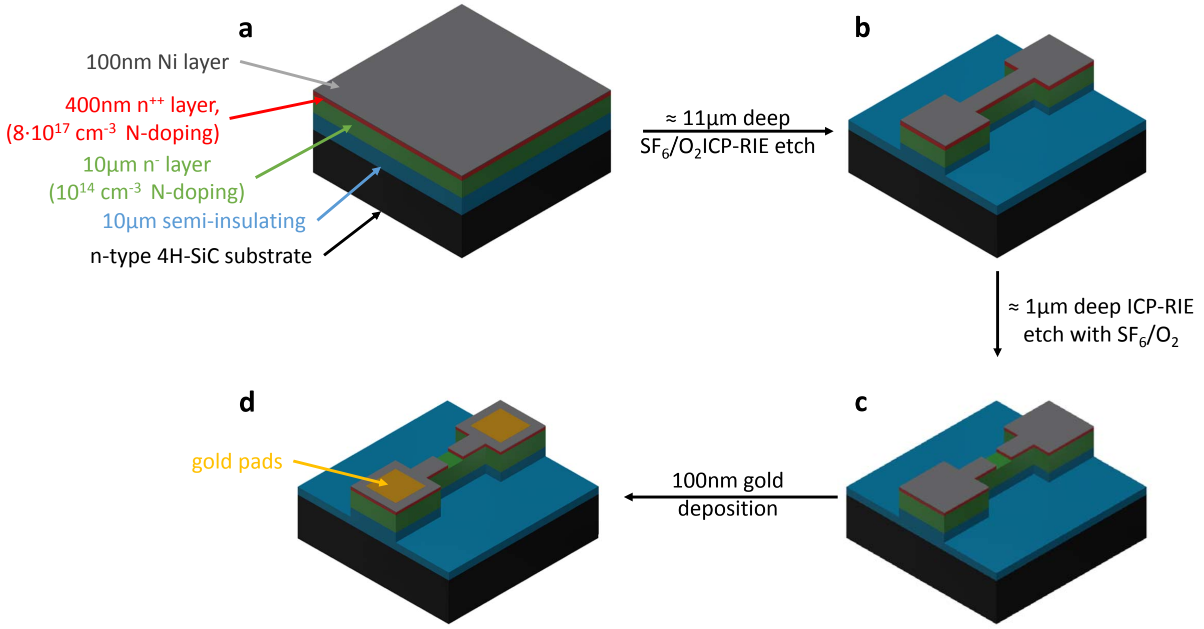

Supplementary Figure 3. Device structure and schematic representation of the device fabrication process. **a** Initial sample layout. **b** Device geometry after the first deep etch step, isolating the devices from others. **c** Removal of the n^{++} layer between the contacts to allow optical access. **d** Final device configuration with two gold pads for wirebonding.

Supplementary Figure 3**a** shows the sample layout before structuring. While the Ni layer serves as a Schottky contact in the final device, it is also used as an etching mask during

the fabrication process. All dry etching steps are performed using an ICP-RIE with SF_6/O_2 gas mixture, which realizes high etching selectivity of SiC over Ni. In the first step, all Ni outside the device region is removed. The sample is then plasma-etched ≈11 µm deep, which removes the n^{++} layer and the n^{-} layer and stops in the semi-insulating layer (Supp. Fig. 3**b**). During this step, the device region is protected by the residual Ni layer. Subsequently, Ni is removed in a rectangular region between the two contact pads of the device. This allows to etch the n^{++} layer in this region by a short SF_6/O_2 ICP-RIE plasma step. The etching depth is chosen to be roughly twice the thickness of the n^{++} layer, ensuring that it is completely removed. Finally, gold pads are deposited on both contacts for wirebonding, resulting in the final device as depicted in Supp. Fig. 3**d**.

Structures for photocurrent detection are usually available as Schottky type, p-i-n or p-n junctions. Here, we use a metal-semiconductor-metal type device, as it does not need p-type doping. This would have required either ion implantation doping (introducing other defects) for lateral devices or a more complex device geometry, as optical access to the diode structure is more difficult to achieve in vertical diodes. Note that the detected very small currents can be further diminished by the high resistivity of transparent conductive layers. The n^{++} structure is needed to form better contact to the metallization and block the depletion layer from the Schottky contact not to affect the Fermi level of the n^{-}- region. The n^{-}- region itself fixes the Fermi level so that V_{Si} is in the right charge state and contains the optically active volume. As diodes also act as detectors for RF and MW signals, we refrained from using interdigit finger structures and minimized the device footprint in order to reduce capacitive RF coupling.

Supplementary Note I: I–V Characteristics of the device used

The I–V characteristics of the device have been measured before and after electron irradiation. Note that these were measured on different experimental setups. For the measurement before irradiation, a source measure unit (SMU, Keithley, 487) with a manual probe station was used. Measurements after irradiation were performed with the PDMR setup using a SMU (Keithley, 2636B). The sample was mounted on a PCB sample holder. Connections between sample and PCB were wirebonded.

As shown in Supp. Fig. 4**a**, the device shows rectifying behavior at positive and negative

bias conditions. Thus, the contacts are assumed to be Schottky type. Then the sample has been irradiated by electrons with a dose of $1 \times 10^{17}\,\mathrm{cm}^{-2}$ and an energy of $2\,\mathrm{MeV}$. The measurement of the irradiated device clearly shows over 2 orders of magnitude less conductivity compared to the non-irradiated device (see Supp. Fig. 4**b**), which we attribute to the doping compensation due to irradiation-induced defects[1,2]. The carbon vacancy is known to be an efficient electron trap[3] and created by electron irradiation, rendering it the most likely candidate. However, as the temperature needed to anneal out V_C is higher than the annealing temperature of V_{Si}^- [4,5], reduction of this effect can only be achieved by more specific defect creation in the first place or by adapting known post treatment processes[6] in the future to preserve V_{Si}^-.

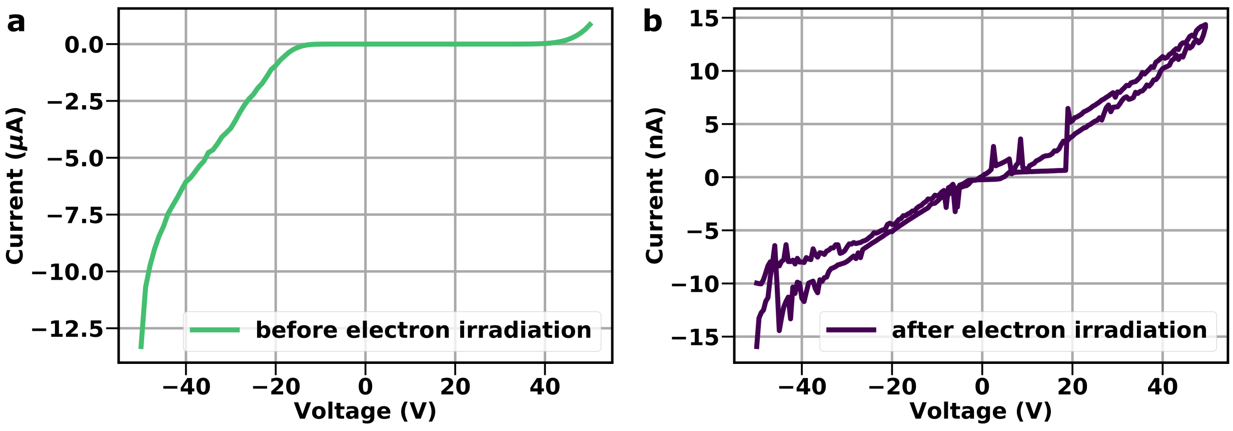

Supplementary Figure 4. I–V characteristics of the device. **a** Before electron irradiation. **b** After electron irradiation.

Supplementary Note II: Position mapping of PDMR signal

To map the position of the obtained PDMR signal to the device structure, we utilize laser scanning. Thereby we subsequently acquire PDMR signal and fluorescence emission of the V_{Si}^- ensemble. The fluorescence locates the device within the given scan range of 100 µm as shown in Supp. Fig. 5. Because the optical detection is performed without spatial filtering, a slight offset in position may exist.

All measurements in the main text have been performed at a fixed depth for consistency. Cross sections of photocurrent and PDMR signals are given in Supp. Fig. 6. The vertical z-slice shows that both photocurrent and PDMR amplitude are dependent on the focal

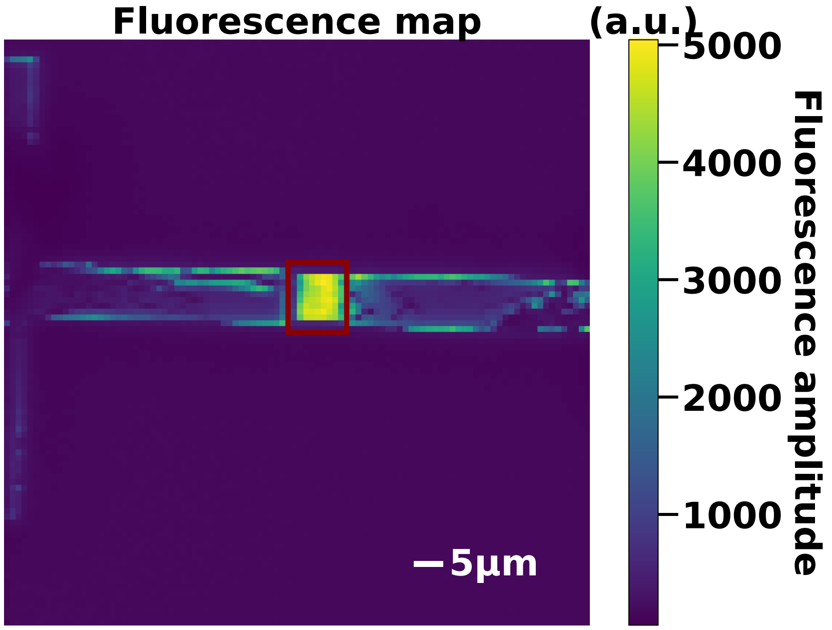

Supplementary Figure 5. Fluorescence detected xy-scan of the device. The rectangular aperture is clearly visible. By this we identify the measurement positions shown in main text in Fig. 1**b**,**d** and Fig. 3**d**.

position. The horizontal xy-slices show a thin strip (marked orange) of effective photocurrent generation that evolves to a larger area when defocusing (marked red). When the focus is inside the device, we do not find a PDMR signal. We attribute this finding to a small excitation volume, which results in a too small number of defects involved in the PDMR process. As increasing the excitation area, we pick up a measureable PDMR signal. However, due to the decrease in laser power density, the signal does not saturate. At the moment, it is unclear to us why this process only appears at the center of the device. A convolution of excitation volume and active area should be the expected result.

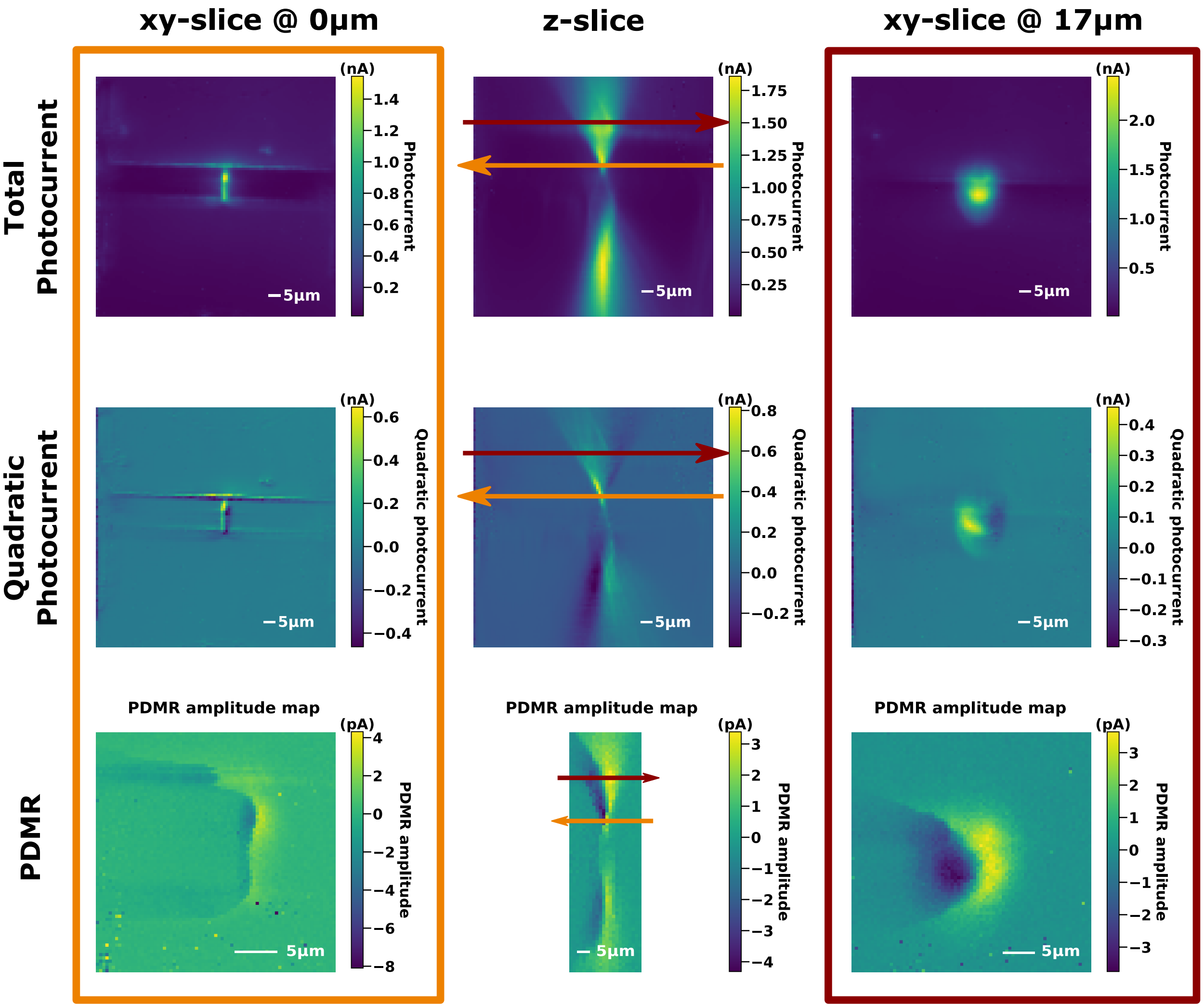

Supplementary Figure 6. Photocurrent, two-photon contribution to photocurrent and PDMR amplitude maps recorded at +20 V bias. Orange and red lines in center z-slices indicate focal position for on-focus (orange) and out-of-focus (red) recorded maps.

Supplementary Note III: Parameter dependencies: Bias voltage, laser power and RF power

Fitting a single resonance peak in a PDMR measurement to the $m_s = +1/2 \leftrightarrow +3/2$ transition gives information on the signal amplitude, linewidth, resonance frequency and the offset signal. The offset signal is mainly caused by stray RF field. Supplementary Figure 7 compares the dependency of ODMR and PDMR signal, on bases of amplitude, linewidth and resonance frequency when bias, laser power and RF power are changed. Note that although

measurements have been performed under the same conditions for ODMR and PDMR, a slight offset in detected volume is possible (see discussion in main text and Supplementary Discussion 6). PDMR signals at small laser or RF powers as well as small bias voltages show low SNR and thus larger error bars. The bias voltage does not affect the ODMR signals. On the other hand, the PDMR amplitude increases monotonically without saturating. The applied bias determines the charge extraction efficiency, and larger bias is expected to increase the measured signal amplitude. Note that linewidth for ODMR and PDMR are similar. A small shift of resonance frequency dependent on bias is visible for PDMR (see Supp. Fig. 7**c**), but not in ODMR. This shift appears in the low bias regime in which the SNR is low. At this moment, we do not have an explanation for this behavior. The incident laser power does change neither linewidth nor resonance frequency. However, the fitted peak amplitude increases for both ODMR and PDMR. This observation is consistent with the suggested mechanism for the PDMR for the V_{Si}^- in SiC because stronger optical excitation will enhance the photo-ionization probability (see Fig. 1**a-c** of the main text). Note that laser power dependence suggests a saturation behaviour for ODMR, while no saturation could be achieved in the PDMR case.

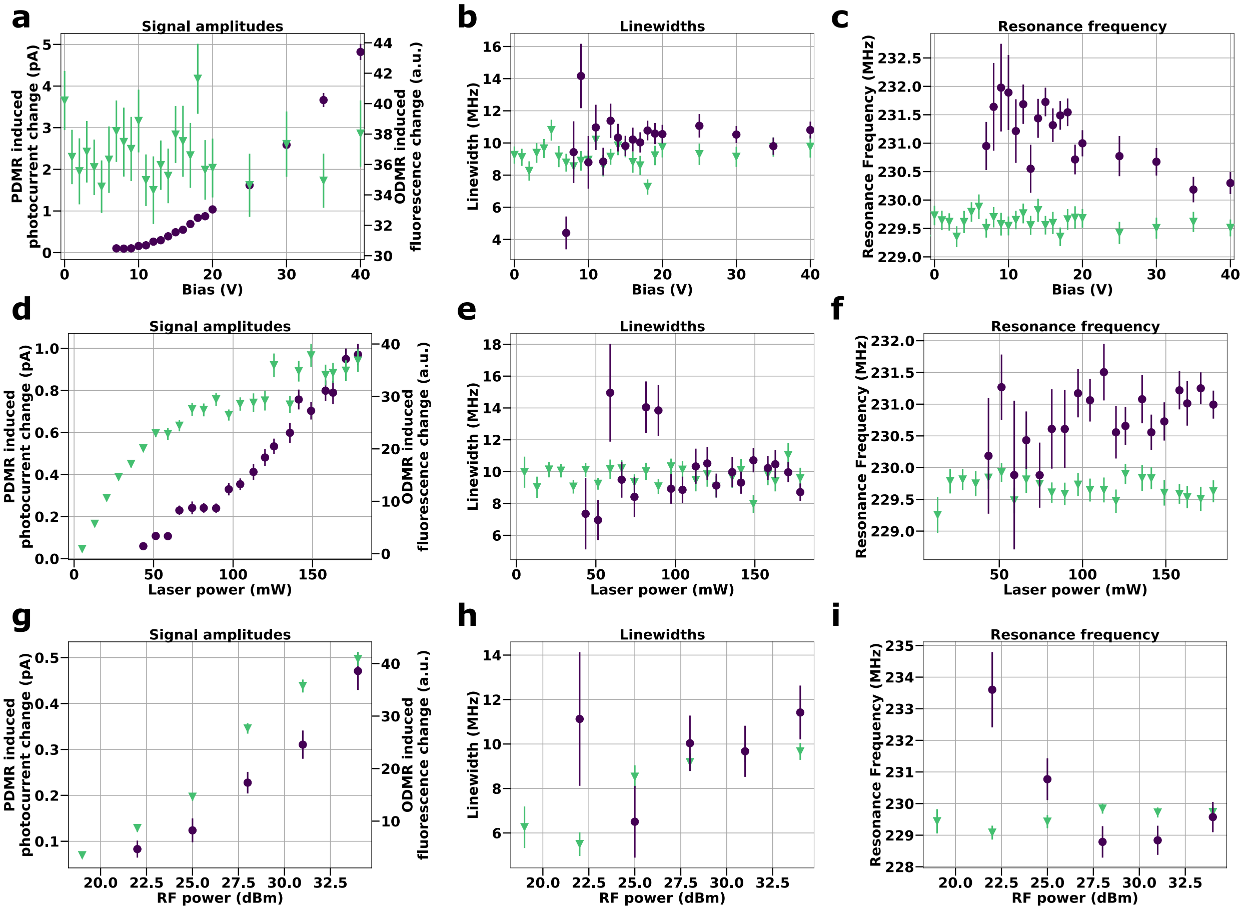

Supplementary Figure 7. Parameter dependence comparison for ODMR (green) and PDMR (purple). Data points are obtained from Gaussian peak fit. Error bars are obtained from standard error of least-square fitting, neglecting the noise contribution at each measurement point. Meausurement conditions: Bias +20 V, 178.5 mW laser and 34 dBm RF power, 223 MHz RF frequency. **a-c** Fitted amplitude, linewidth and resonance frequency dependent on applied bias voltage. **d-f** Fitted amplitude, linewidth and resonance frequency dependent on incident laser power. **g-i** Fitted amplitude, linewidth and resonance frequency dependent on B_1 field strength.

Larger RF power increases linewidth of the magnetic resonance due to power broadening. In case of PDMR, a larger shift in resonance frequency is only visible for the point at smallest RF power at which signal is still picked up in PDMR. At this data point, the signal has very low amplitude. The amplitudes of the PDMR and ODMR signals increase with larger RF power. Assuming inhomogeneous broadening, increasing the RF power increases the excitation bandwidth and thus more defects contribute to the signal. No saturation of the microwave transition is visible. Note that the point of highest applied RF power is already

measured in the RF amplifier compression regime.

Supplementary Note IV: Discussion on PDMR contrast, SNR and sensitivity

In a typical ODMR experiment, the contrast c is defined as the spin-dependent fluorescence change Δ_{PL} at resonance to the off resonance fluorescent signal $\mathrm{PL}_{\mathrm{BG}}$ (baseline):

$$c = \Delta_{\mathrm{PL}}/\mathrm{PL}_{\mathrm{BG}}. \tag{1}$$

When the detected signal change increases the signal (as in ODMR of $\mathrm{V}_{\mathrm{Si}}^{-}$), such a definition can lead to contrast larger than 100% and results in difficulties to compare contrasts for positive and negative signal amplitudes. As for PDMR the sign of the amplitude can be different, a more versatile definition is needed.

Based on the original definition of contrast, we further extend this definition by comparing the amplitude to the maximum acquired signal as follows:

$$c = \mathrm{A}/\max(|\mathrm{BG}|, |\mathrm{BG} + \mathrm{A}|). \tag{2}$$

Here, BG is the fluorescence background or dc offset of the PDMR signal and A is the ODMR or PDMR amplitudes at measurement condition far away from resonance. By this definition, the maximum achievable contrast by fluorescence is limited to 100%, resulting in a more meaningful quantity.

In order to measure the correct dc background, we use an oscilloscope in parallel to the lock-in amplifier and measure the mean value within a 0.5 s integration window to get the dc offset for each magnetic field point.

We then take the mean value of these points as dc offset. In this recorded data, the PDMR amplitude is also contained within the data for on-resonance points.

As in the case of $\mathrm{V}_{\mathrm{Si}}^{-}$ the PDMR and ODMR amplitudes are very small compared to the dc offset (≈4 orders of magnitude), the contribution is negligible. The same argument holds for the difference between definitions in Supporting Eq. 1 and Supporting Eq. 2. Thus in case of low relative amplitudes, our extended definition of contrast is still comparable to prior work.

Next, we analyze the dependence of contrast and SNR on the experimental conditions. To correct for differences in measurement time we normalize the SNR to $t_{\mathrm{norm}} = 3600\,\mathrm{s}$. The value for this time-normalized $\mathrm{SNR}_{\mathrm{norm}}$ is then calculated by

$$\mathrm{SNR}_{\mathrm{norm}} = \mathrm{SNR}\sqrt{t_{\mathrm{norm}}/t_{\mathrm{meas}}}, \tag{3}$$

where t_{meas} is the total measurement time and SNR the signal-to-noise ratio calculated by dividing the fitted amplitude by the obtained standard deviation noise (see Supp. Fig. 9 and following calculation of dc magnetic field sensitivity). In ODMR measurements, the contrast and $\mathrm{SNR}_{\mathrm{norm}}$ do not depend on the bias, while they do for PDMR as shown in Supp. Fig. 8**a** and **b**. In PDMR, both contrast and $\mathrm{SNR}_{\mathrm{norm}}$, are improved for larger bias voltages. We attribute this to a better extraction efficiency of free electrons and holes in case of PDMR. In terms of contrast a saturating behavior is visible for larger biases. As can be seen in Supp. Fig. 7**a**, the amplitude is still increasing, thus the dc offset must increase more quickly then the signal in this regime. In case of laser power dependence, we see that ODMR contrast decreases for high laser powers, while the PDMR contrast still increases (see Supp. Fig. 8**c**). The time-normalized SNR shown in Supp. Fig. 8**d** saturates for ODMR, whereas SNR in the PDMR case still increases with laser power. If we vary the applied RF power, a clear rise in contrast is visible in both measurement techniques, as depicted in Supp. Fig. 8**e**. $\mathrm{SNR}_{\mathrm{norm}}$ is increasing for both PDMR and ODMR with applied RF power. However, larger RF power leads to larger noise for PDMR due to the RF coupling. The gain in $\mathrm{SNR}_{\mathrm{norm}}$ is thus bigger for ODMR than for PDMR.

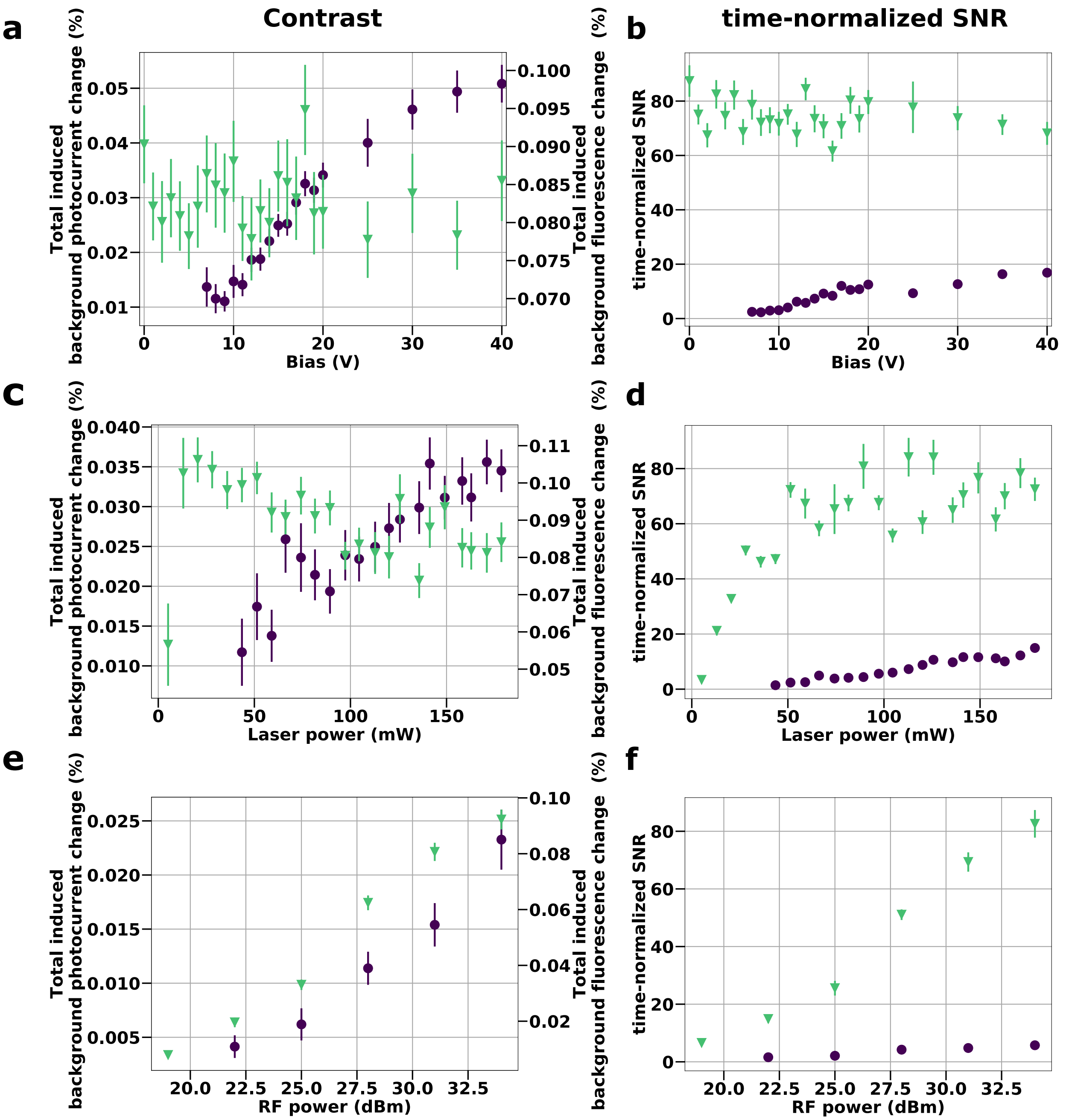

Supplementary Figure 8. DC referred contrast and time-normalized (3600 s) SNR dependent on measurement conditions for ODMR (green) and PDMR (purple). Error bars are obtained by standard error of least-square fitting and error propagation. Meausurement conditions: Bias +20 V, 178.5 mW laser and 34 dBm RF power, 223 MHz RF frequency. **a-b** Bias dependence of contrast and SNR. **c-d** Laser power dependence of contrast and SNR. **e-f** B_1 field strength dependence of contrast and SNR.

In the following, we calculate the dc magnetic field sensitivity. For this we use the ODMR and PDMR data shown in Fig. 3**b** in the main text. The sensitivity is given by comparing the signal power to the noise spectral power. We estimate the noise by using data points at least 3σ apart from the resonance (see Supp. Fig. 9) and calculating the standard deviation of these data points. This way, we extract a noise level of 70 fA. Signal-to-noise ratio is then obtained by dividing the PDMR resonance amplitude by the noise level. The measurement time per point is 25 s for this PDMR measurement, resulting in a noise spectral density of $350\,\mathrm{fA\,Hz^{-1/2}}$. The slope of the Gaussian peak is maximum at σ distance, related to the FWHM by $\mathrm{FWHM} = 2\sqrt{2\ln 2}\sigma$ (see Supp. Fig. 9). Thus the position of the steepest slope can be found by the amplitude and FWHM of the fitted peak and we find a maximum slope of $1.3\,\mathrm{nA\,T^{-1}}$. From this, we calculate a magnetic field sensitivity of $253\,\mathrm{\mu T\,Hz^{-1/2}}$. For the ODMR, we find a noise level of 290 µV within a measurement time of 2.8 s per point. With a slope of $21\,\mathrm{mV\,T^{-1}}$ we calculate the sensitivity to be $23\,\mathrm{\mu T\,Hz^{-1/2}}$. Many quantum applications (such as metrology, computation and communication) impose high-speed operation on qubits and quantum memories. We approximate a bandwidth of about 500 kHz-1 MHz based on the initialization (ISC cycle: 200-300 ns) rate and achieved Rabi frequency (1-1.5 MHz), which is similar to ODMR.

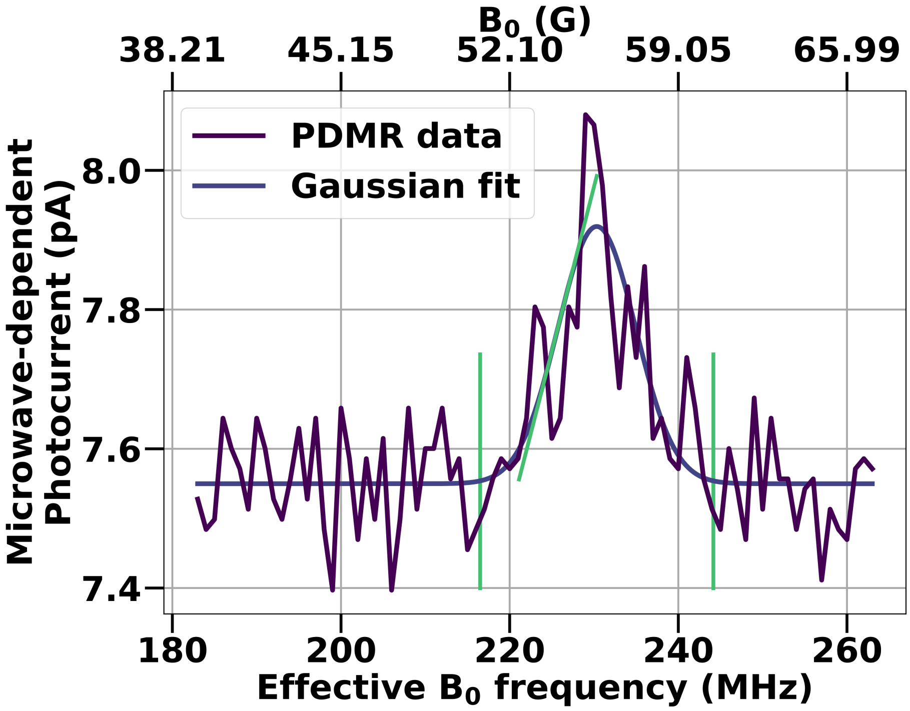

Supplementary Figure 9. Extraction of magnetic field sensitivity and SNR for PDMR. The slope is extracted from the fitted Gaussian at σ distance from the resonance peak. Noise is determined as standard deviation of measurement points outside of area marked with vertical green lines.

Note that we have not reached the saturation of PDMR signal because of the limitation of the laser power. Bias and RF power dependence also promise further improvement in SNR and sensitivity. In addition, while the PDMR contrast is $\approx 1/10$ of the ODMR contrast, in theory, comparable values might be achievable, as the underlying ISC process is the same. Thus the SNR, sensitivities and contrast given in the main text and the supporting information have to be understood as a lower achievable limit.

Supplementary Note V: Hahn echo measurements

In order to show that the photo-electrical readout does not hinder spin measurements and has no major detrimental effect on coherence, Hahn echo measurements have been performed using both optical and electrical readout. As known from theory and experiment, the ^{29}Si and ^{13}C nuclear spins baths present in natural abundance in the used sample are not well decoupled from each other at low magnetic fields[7–9]. This leads to electron spin envelope echo

modulation (ESEEM), resulting in a short decay at very low magnetic fields. Increasing the magnetic field strength will increase visible revivals of the spin signal until a strong enough magnetic field is present to decouple the two spin baths. Supporting Figure 10**a** shows the Hahn echo response obtained at $B_0 \approx$ 54 G. Here, the field strength is not strong enough to dilute the nuclear spin bath (see also Supp. Ref. [8] Fig. 4**a**, note different definition of x-axis) and a short decay is visible. As can be seen, the echo signals detected by ODMR are not influenced by applied bias voltages, *i.e.*, the same results are observed at 0 V and at 20 V. Data is fitted by a Gaussian decay with signal decrease to 1/e defined as decay time (decay times from fit: ODMR 0 V: 7.8 ± 0.3 µs, ODMR 20 V: 7.8 ± 0.3 µs, PDMR 20 V: 7.5 ± 0.4 µs). As the measurement scheme under the latter condition is the same as for the electrical readout, the data suggests that the applied bias does not alter the coherence properties under these conditions. The Hahn echo obtained using PDMR technique shows very similar behavior with a minimal shorter decay time.

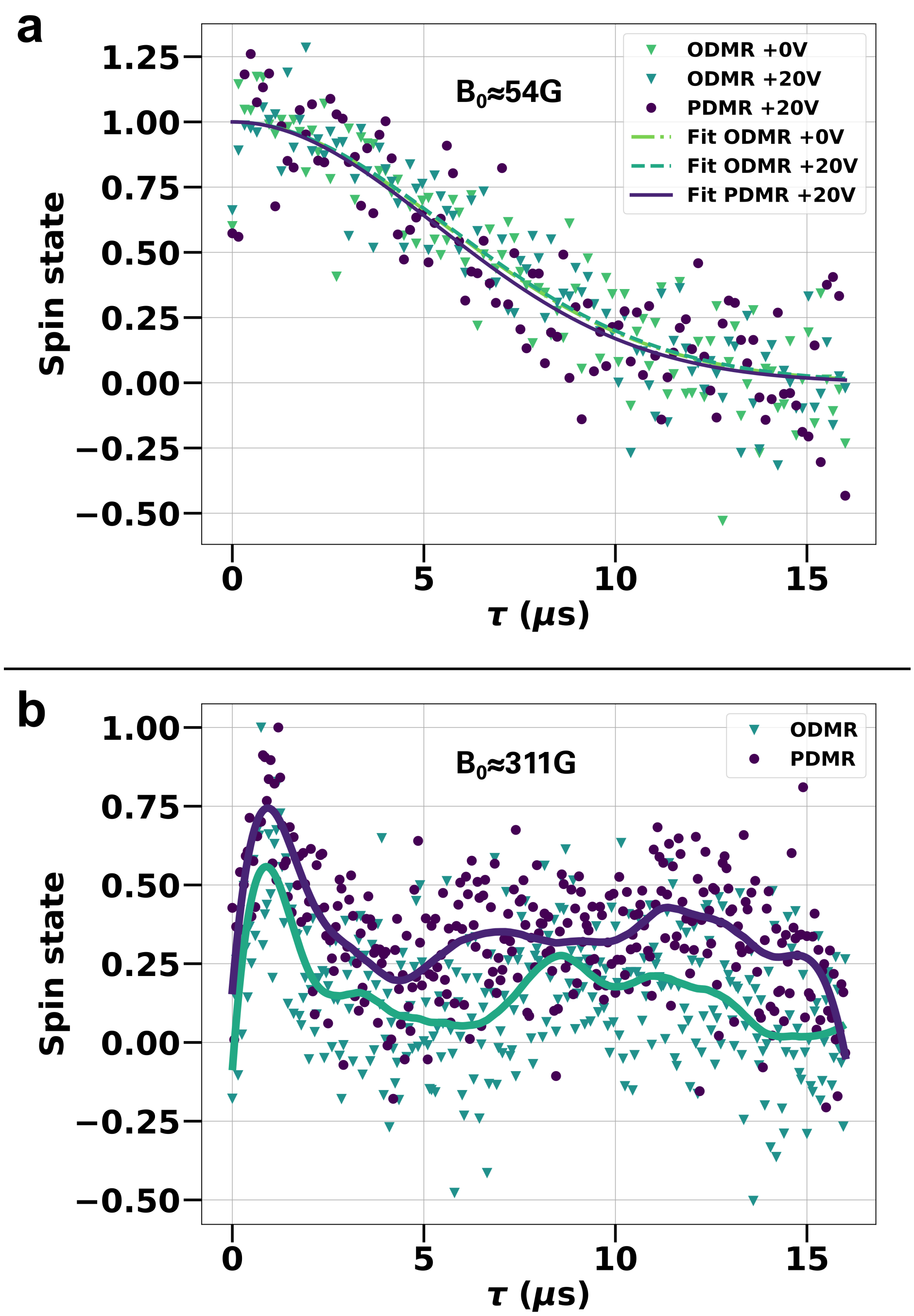

Supplementary Figure 10. Hahn echo measured at different magnetic field using ODMR and PDMR techniques. **a** Hahn echo response at $B_0 \approx$ 54 G showing quick decay due to ESEEM. Applied bias of 0 V and 20 V does not affect signal in optical detection. Fit functions are Gaussian, data normalized to fit. **b** Echo response at $B_0 \approx$ 311 G and 20 V shows preserved coherence visible as signal revivals. Lines are guide-for-the-eye obtained by filtering. Data normalized to maximum.

After placing a permanent magnet above the sample and coarse alignment of this magnetic field to the c-axis of the crystal, we achieve a higher magnetic field of $B_0 \approx 311\,\mathrm{G}$. At this field strength, revivals of the spin echo are expected. As can be seen in Supp. Fig. 10**b**, the revivals are visible indeed. As guide for the eye, the noisy signal is filtered by a 3rd order Savitzky-Golay filter with 49 samples window-length two times in series. Due to the low SNR, the maximum duty cycle is limited and data can only be provided up to 16 µs. The envelope modulation and limited achievable free evolution time hinder extraction of a T_2 coherence time. However, after 14 µs the echo signal appears still intact. The data of ODMR and PDMR do not agree perfectly in the visible signal revivals, which we attribute to slightly different field strength and resonance frequency conditions of both signals. Overall, the fast decay and complex behavior of Supp. Fig. 10**a** and **b**, respectively, resemble the previously shown observation of ESEEM by others[8].

Supplementary Note VI: Local magnetic fields variations inside the device

In order to check for magnetic fields within the device likely introduced by magnetization of the Ni contacts, we perform ODMR measurements at different depths and x-positions and extract the resonance frequency. The results are shown in Supp. Fig. 11. As the lines can shift a couple of MHz, magnetic field offsets of a few Gauss appear to be plausible in the device structure. This position-dependent magnetic field indicates that there can be a difference in the local external fields between PDMR and ODMR, if both measurements exhibit a slightly shifted position of the detected volume.

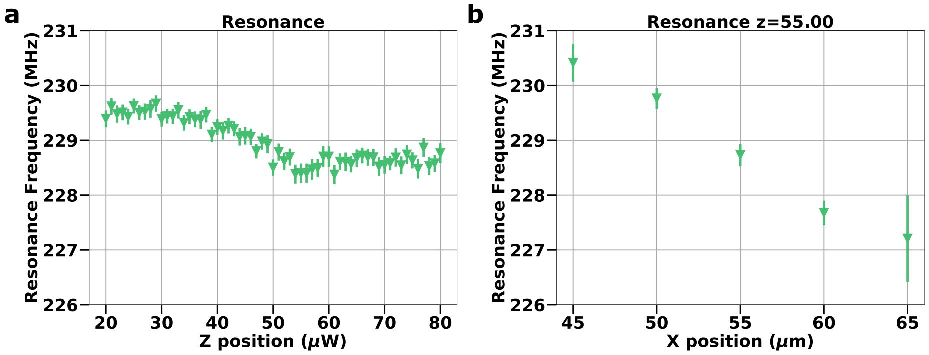

Supplementary Figure 11. Magnetic field variation within device. Shift in ODMR resonance peak position depending on **a** z-position and **b** x-position. Error bars obtained from standard error of least-square fitting.

Supplementary Note VII: Estimation of the number of defect spins addressed

Estimation of the defect spins contributing to the photocurrent signal is performed in the following. Note, that the numbers rely on many assumptions and thus can only give a very rough estimate of minimum current contribution. E.g., we have to assume that the achieved current is saturated, which according to Supplementary Figure 7**a**,**d**,**g** is not the case. From previous work, we expect a defect density on the order of $500\,\mu m^{-3}$ formed by the applied irradiation dose[10]. Note, that the density of active centers can drastically change depending on the exact doping condition or free carrier density. In ODMR, the full focal volume can be considered to contribute to the fluorescence signal. As a two-photon process is needed for PDMR to work, the effective active volume is much smaller, thus reducing the number of defects involved. The intensity distribution of a Gaussian beam is given by

$$I(r,z) = \left(I_0 \frac{1}{1+(z/z_r)^2} e^{\left(-2r^2/w^2(z)\right)} \right)^y, \tag{4}$$

where y is the number of photons involved in the process (non-linearity) and z_r is the Rayleigh length. The beam width $w(z)$ for a Gaussian beam is defined as:

$$w(z) = w_0 \sqrt{1+(z/z_r)^2}, \tag{5}$$

with w_0 the beam waist. We calculate the beam waist from the diffraction limited FWHM for wavelength λ and objective with a certain numerical aperture (NA) given by

$$\mathrm{FWHM} = \frac{\lambda}{2\mathrm{NA}}. \tag{6}$$

This is related to the beam waist by FWHM $= w_0\sqrt{2\ln 2}$. From this, the effective focal volume is given by the volume of the ellipsis where intensity drops to $1/e^2$ and thus of length

$$L_y = 2z_r\sqrt{e^{2/y} - 1} \tag{7}$$

and diameter

$$d_y = 2w_0/\sqrt{y}. \tag{8}$$

The objective used (NA=0.65) thus ideally provides a Gaussian beam with a minimum focal volume of 8 µm^3 at 785 nm in 4H-SiC , ($n_{SiC} = 2.6$), corresponding to ≈ 4000 defects. Assuming a two-photon ionization process, the volume shrinks to 2 µm^3 and thus ≈ 1000 defects contribute to the PDMR signal. With a maximum detected PDMR current of 4 pA (see Fig. 3**d** in main text), this leads to a rough estimation on the current contribution of 4 fA per defect. As this neglects the device electron extraction efficiency, possible internal amplification mechanisms, the unsaturated current and we assume the laser-beam diameter fully matches the back-aperture of the objective, we expect this to be very crude estimates for the focal volumes and minimum current contribution. Thus the calculated numbers should be treated with caution.

Supplementary References

* matthias.niethammer@pi3.uni-stuttgart.de

[1] Ohyama, H. *et al.* Radiation damage of SiC Schottky diodes by electron irradiation. *J. Mater. Sci.: Mater. Electron.* **16**, 455–458 (2005). URL http://link.springer.com/10.1007/s10854-005-2314-4.

[2] Son, N. T., Carlsson, P., ul Hassan, J., Magnusson, B. & Janzén, E. Defects and carrier compensation in semi-insulating 4h-SiC substrates. *Physical Review B* **75** (2007). URL https://link.aps.org/doi/10.1103/PhysRevB.75.155204.

[3] Hiyoshi, T. & Kimoto, T. Elimination of the Major Deep Levels in n- and p-Type 4h-SiC by Two-Step Thermal Treatment. *Applied Physics Express* **2**, 091101 (2009). URL http://stacks.iop.org/1882-0786/2/091101.

[4] Zolnai, Z., Son, N. T., Hallin, C. & Janzén, E. Annealing behavior of the carbon vacancy in electron-irradiated 4h-SiC. *Journal of Applied Physics* **96**, 2406–2408 (2004). URL http://aip.scitation.org/doi/10.1063/1.1771472.

[5] Wang, X. *et al.* Formation and annealing behaviors of qubit centers in 4h-SiC from first principles. *Journal of Applied Physics* **114**, 194305 (2013). URL http://aip.scitation.org/doi/10.1063/1.4832457.

[6] Storasta, L. & Tsuchida, H. Reduction of traps and improvement of carrier lifetime in 4h-SiC epilayers by ion implantation. *Applied Physics Letters* **90**, 062116 (2007). URL http://aip.scitation.org/doi/10.1063/1.2472530.

[7] Yang, L.-P. *et al.* Electron spin decoherence in silicon carbide nuclear spin bath. *Phys. Rev. B* **90**, 241203(R) (2014). URL https://link.aps.org/doi/10.1103/PhysRevB.90.241203.

[8] Carter, S. G., Soykal, Ö. O., Dev, P., Economou, S. E. & Glaser, E. R. Spin coherence and echo modulation of the silicon vacancy in 4H-SiC at room temperature. *Phys. Rev. B* **92**, 161202(R) (2015). URL https://link.aps.org/doi/10.1103/PhysRevB.92.161202.

[9] Seo, H. *et al.* Quantum decoherence dynamics of divacancy spins in silicon carbide. *Nature Communications* **7** (2016). URL http://www.nature.com/articles/ncomms12935.

[10] Widmann, M. *et al.* Coherent control of single spins in silicon carbide at room temperature. *Nat. Mater.* **14**, 164–168 (2015). URL http://www.nature.com/articles/nmat4145.
